# Epigenetic downregulation of Socs2 contributes to mutant N-Ras-mediated hematopoietic dysregulation

**DOI:** 10.1242/dmm.049088

**Published:** 2022-05-06

**Authors:** Xi Jin, Victor Ng, Meiling Zhao, Lu Liu, Tomoyasu Higashimoto, Zheng Hong Lee, Jooho Chung, Victor Chen, Gina Ney, Malathi Kandarpa, Moshe Talpaz, Qing Li

**Affiliations:** 1 Department of Internal Medicine, University of Michigan, Ann Arbor, MI 48109, USA; 2 Department of Pediatrics, University of Michigan, Ann Arbor, MI 48109, USA; 3 Department of Cell and Developmental Biology, University of Michigan, Ann Arbor, MI 48109, USA

**Keywords:** RAS signaling, Jak/Stat signaling, SOCS proteins, Hematopoietic stem cells, Leukemia, Epigenetic regulation

## Abstract

RAS mutations occur in a broad spectrum of human hematopoietic malignancies. Activating Ras mutations in blood cells leads to hematopoietic malignancies in mice. In murine hematopoietic stem cells (HSCs), mutant N-Ras^G12D^ activates Stat5 to dysregulate stem cell function. However, the underlying mechanism remains elusive. In this study, we demonstrate that Stat5 activation induced by a hyperactive *Nras* mutant, *G12D*, is dependent on Jak2 activity. Jak2 is activated in *Nras* mutant HSCs and progenitors (HSPCs), and inhibiting Jak2 with ruxolitinib significantly decreases Stat5 activation and HSPC hyper-proliferation *in vivo* in *Nras^G12D^* mice. Activation of Jak2-Stat5 is associated with downregulation of Socs2, an inhibitory effector of Jak2/Stat5. Restoration of Socs2 blocks *Nras^G12D^* HSC reconstitution in bone marrow transplant recipients. *SOCS2* downregulation is also observed in human acute myeloid leukemia (AML) cells that carry RAS mutations. RAS mutant AML cells exhibited suppression of the enhancer active marker H3K27ac at the *SOCS2* locus. Finally, restoration of *SOCS2* in RAS mutant AML cells mitigated leukemic growth. Thus, we discovered a novel signaling feedback loop whereby hyperactive Ras signaling activates Jak2/Stat5 via suppression of Socs2.

## Abstract (precis)

**Summary:** Jak2/Stat5 is often considered to be parallel to or upstream of Ras signaling. We have discovered a novel signaling feedback loop whereby hyperactive Ras signaling activates Jak2/Stat5 via suppression of Socs2.

## INTRODUCTION

Activation of oncogenic Ras signaling is one of the most prevalent mechanisms in human cancer pathogenesis. RAS genes encode a family of 21 kDa proteins that transduce cell proliferation and survival signals from extracellular stimuli to intracellular effector pathways, thus controlling cell fate. Somatic mutations of RAS genes are frequently detected in hematopoietic malignancies (Ney et al., 2020; Ward et al., 2012). We have shown previously that the gain-of-function mutation *Nras^G12D^* drives initiation of indolent myeloproliferative disorders in mice (Li et al., 2011; Wang et al., 2011). At the pre-leukemic stage, N-Ras^G12D^ promotes hematopoietic stem and progenitor cell (HSPC) clonal expansion by increasing both cell proliferation and self-renewal potential (Li et al., 2013). This bimodal effect builds up a reservoir of mutant cells that outcompete wild-type cells to facilitate the acquisition of additional mutations for transformation into leukemia. The effects of N-Ras^G12D^ in hematopoietic stem cells (HSCs) at least partially depends on activation of Stat5 signaling, and knockout of *Stat5* in *Nras^G12D^* mice abolishes the proliferative and reconstitution advantage of the *Nras* mutant HSCs (Li et al., 2013). Although hyperactivation of Stat5 signaling has been implicated in different leukemia types (Dorritie et al., 2014), the mechanism of how mutant N-Ras activates Stat5 signaling is not clear.

The suppressor of cytokine signaling (Socs) proteins are transcription targets of Stat proteins and are also known as negative-feedback effectors that prevent overt activation of Jak-Stat, which may cause uncontrolled cell proliferation (Durham et al., 2019). Socs2 is best known as a key regulator in controlling growth hormone (GH)-Stat5 signaling, and *Socs2*-deficient mice are gigantic (Greenhalgh et al., 2002; Metcalf et al., 2000). At steady state, Socs2 is dispensable for basal hematopoiesis and HSC competitiveness (Hansen et al., 2013; Vitali et al., 2015). However, under myelopoietic stress conditions induced by 5-fluorouracil (5-FU), Socs2 deficiency promotes expansion of differentiated hematopoietic populations, induces transient increase of HSC proliferation and eventually leads to HSC exhaustion in serial bone marrow transplantation models (Hansen et al., 2013). Dysregulated expression of *SOCS2* has been reported in several hematopoietic malignancies (Hansen et al., 2013; Janke et al., 2014; Laszlo et al., 2014; Nguyen et al., 2019; Puigdecanet et al., 2008; Vitali et al., 2015), with some showing association of *SOCS2* expression with poor prognosis of the disease (Laszlo et al., 2014; Nguyen et al., 2019). The role of Socs2 in disease pathogenesis and management is largely dependent on disease type, on upstream mutations causing Socs2 dysregulation and on cell populations being investigated.

In this study, we found that oncogenic N-Ras^G12D^ specifically upregulated Jak2-Stat5 signaling in murine HSPCs through suppression of Socs2 expression. Hyperactivation of Nras signaling led to a profound effect on suppressing Socs2 expression in hematopoietic stem and progenitor cells (HSPCs). Restoration of Socs2 expression significantly impaired blood cell reconstitution of the *Nras^G12D^* mutant HSCs in transplant recipients. Further investigation in human AML cells revealed that mutant *NRAS* is associated with downregulation of the active enhancer marker H3K27ac at the *SOCS2* locus and significant reduction of *SOCS2* expression. Importantly, restoration of Socs2 expression significantly mitigated growth of *NRAS* mutant leukemia cells. Thus, our data suggest a novel role for oncogenic Ras in activating Jak2/Stat5 signaling via epigenetic regulation of Socs2 in both pre-leukemic HSCs and fully developed leukemic cells.

## RESULTS

### N-Ras^G12D^ activates Jak2-Stat5 axis to promote HSPC hyper-proliferation

**Fig. 1.**
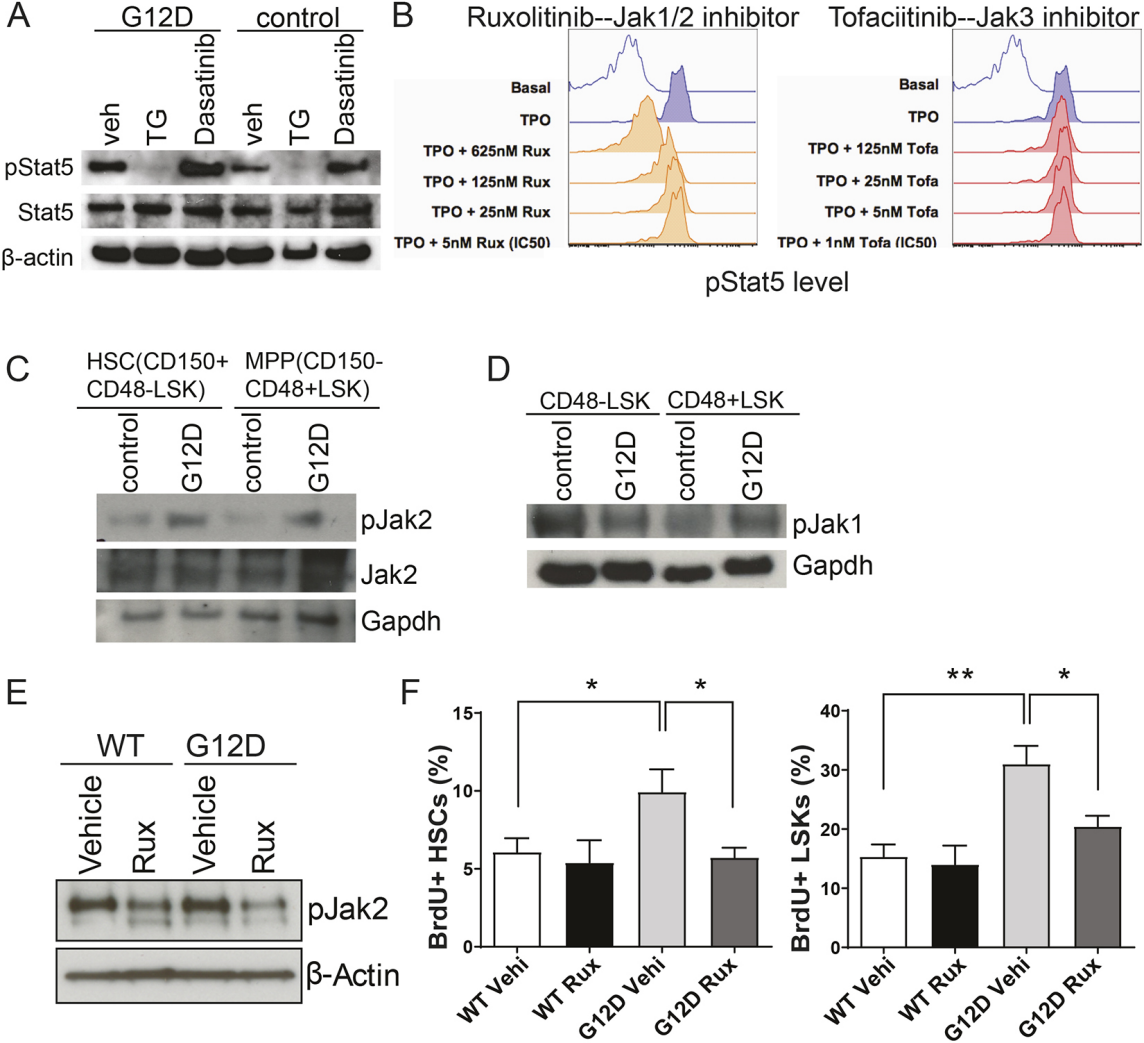
**N-Ras^G12D^ activates the Jak2-Stat5 axis to promote HSPC hyper-proliferation.** (A) Western blot analysis of pStat5 in purified wild-type or *Nras^G12D/+^* LSKs treated with either vehicle, the pan-Jak inhibitor TG101348 or the Bcr/Abl and Src inhibitor dasatinib (500 nM for 30 min in the presence of 10 ng/ml SCF and TPO). (B) FACS analysis of pStat5 in *Nras^G12D/+^* CD48^−^LSKs (HSCs and MPPs) pre-treated with the Jak1/2 inhibitor ruxolitinib or the Jak3 inhibitor tofacitinib, followed by TPO stimulation (10 ng/ml for 15 min). (C) Western blot analysis of pJak2 at steady state in primitive HSCs and MPPs. (D) Western blot analysis of pJak1 at steady state in CD48^−^LSKs (HSCs and MPPs) and CD48^+^LSKs (multi-lineage progenitors). (E) Western blot analysis of pJak2 (Tyr221) in whole bone marrow cells from wild-type or *Nras^G12D/+^* mice treated with vehicle or ruxolitinib (30 mg/kg, twice a day for 7 days). (F) Frequency of BrdU incorporation in HSCs and LSKs from ruxolitinib-treated mice (E) after a 24-h BrdU pulse (*n*=5 per group). Data are mean±s.d. Unpaired two-tailed Student's *t*-test was used to assess statistical significance. *P≤0.05, **P≤0.01.

Our previous findings have demonstrated that Stat5 is required for N-Ras^G12D^-induced increase in HSPC proliferation and reconstitution (Li et al., 2013). Here, we sought to explore how oncogenic N-Ras activates the Stat5 signaling pathway, given that Stat signaling is considered to be an upstream or parallel pathway of Ras signaling. To do this, we generated *Mx1-cre^+^; Nras^LSL-G12D/+^* mice using a previously described *Nras^G12D^* allele in which the oncogenic *G12D* mutation was expressed endogenously from the *Nras* locus downstream of a floxed ‘STOP’ cassette that represses transcription (Li et al., 2013, 2011). To induce deletion of the ‘STOP’ cassette and activate the mutant *G12D* allele in hematopoietic tissues, we administered three doses of pIpC over a 5-day period (at a dose of 0.5 µg/g body mass) to 6- to 10-week-old *Mx1-cre^+^; Nras^LSL-G12D/+^* mice (referred to hereafter as *Nras^G12D^* or *Nras* mutant mice). The mice were then analyzed for a minimum of 2 weeks after pIpC treatment and paired with sex- and age-matched controls that lacked a *Cre* allele (referred to hereafter as control). Stat5 can be activated by the non-tyrosine kinase family Jak or by Src/Abl (Jatiani et al., 2010); therefore, we treated purified wild-type or *Nras^G12D^* mutant Lineage^−^ Sca1^+^Kit^+^ cells (LSKs; containing HSCs and early progenitor cells; Fig. S1) with either a Jak inhibitor, TG101348, or a Src/Abl inhibitor, dasatinib. Without inhibitor treatment, LSKs from *Nras^G12D^* mutant mice displayed a higher level of pStat5 compared with wild type (Fig. 1A). With the treatment of TG101348 but not dasatinib, phosphorylation of Stat5 was completely blocked (Fig. 1A), suggesting that Jak activity is required for N-Ras^G12D^-mediated Stat5 activation. Of note, at a concentration of 500 nM, TG101348 demonstrates inhibitory activity on JAK1 (IC50=105 nM), JAK2 (IC50=6 nM), JAK3 (IC50=∼1 µM) and TYK2 (IC50=405 nM) (Wernig et al., 2008). To determine which Jak isoform is required for pStat5 activation, we next treated LSK cells with a specific Jak1/2 inhibitor, ruxolitinib, or a Jak3 inhibitor, rofacitinib (Fig. 1B). Phospho-FACS revealed that N-Ras^G12D^ requires Jak1/2 but not Jak3 for thrombopoietin (TPO)-induced Stat5 activation in LSKs (Fig. 1B). To further investigate whether Jak1 or Jak2 is dysregulated in *Nras* mutant HSPCs, the levels of phosphorylated Jak1 and Jak2 were measured in FACS-purified CD48^−^LSK cells (Fig. S1), which contain HSCs and multi-potent progenitors (MPP). An elevated level of Jak2 phosphorylation (Fig. 1C, Fig. S2A), but not Jak1 phosphorylation (Fig. 1D, Fig. S2B), was observed in *Nras* mutant HSPCs compared with wild type, indicating that N-Ras^G12D^-induced Stat5 activation is mainly dependent on Jak2 activation in HSPCs.

We then sought to explore the effect of blocking Jak2 on N-Ras^G12D^-mediated HSPC hyper-proliferation. *Nras* mutant or control mice were treated with either ruxolitinib (30 mg/kg body mass) or vehicle twice a day for 7 days. Mice were then injected with bromodeoxyuridine (BrdU; 200 mg/kg) 24 h prior to analysis and were kept on BrdU water (1 mg/ml) to determine *in vivo* HSPC proliferation. BrdU incorporation decreased significantly in ruxolitinib-treated versus vehicle-treated *Nras^G12D^* HSCs and LSKs, suggesting that Jak2 is essential for *Nras^G12D^*-mediated HSPC hyper-proliferation (Fig. 1E,F).

Next, we asked whether loss of Jak2 activity impairs *Nras^G12D^* HSPC competitiveness and reconstitution. To address this, we crossed *Mx1-cre^+^; Nras^LSL-G12D/+^* mice (Li et al., 2011) with *Jak2* conditional knockout mice (Krempler et al., 2004). As homozygous deletion of *Jak2* in hematopoietic cells has been shown to severely impair HSC function and lead to fatal bone marrow failure (Akada et al., 2014), we generated *Nras^G12D^* mice with heterozygous deletion of *Jak2*. To assess HSPC competitiveness, we transplanted 5×10^5^ bone marrow (BM) cells from CD45.2 donor wild-type control, *Nras^G12D^*, *Jak2^+/−^* and *Jak2^+/−^;* *Nras^G12D^* double mutant mice along with equal numbers of CD45.1 wild-type BM cells into lethally irradiated CD45.1 recipient mice. Transplant recipients were then bled every 4 weeks to measure the donor-derived myeloid (Mac1^+^Gr1^+^) and lymphoid (B220^+^ or CD3^+^) cells in peripheral blood (as a percentage of CD45.2-positive cells). Consistent with previous data (Li et al., 2013), recipients of *Nras^G12D^* cells exhibited significantly increased donor reconstitution in all lineages compared with wild-type control. Deletion of one copy of *Jak2* did not reduce the level of *Nras^G12D^* HSPC competitiveness (Fig. S3). This suggests that either the remaining allele of *Jak2* is sufficient to sustain its activity or that other Jaks provide compensatory effect upon *Jak2* heterozygous deletion.

### N-Ras^G12D^ suppresses the negative regulator of Jak-Stat signaling in HSPCs

**Fig. 2.**
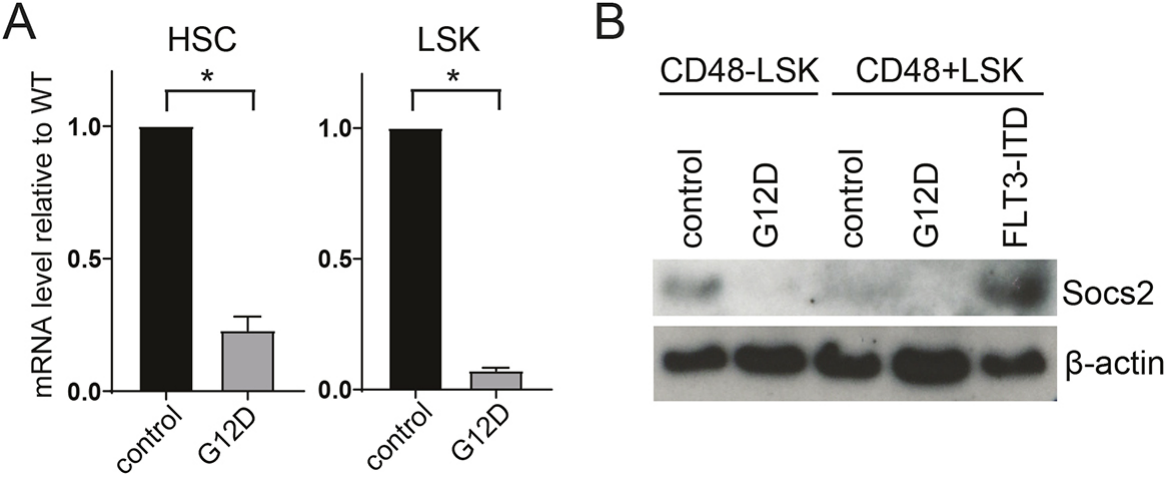
**Oncogenic N-Ras^G12D^ suppresses *Socs2* gene expression in HSPCs.** (A) Quantitative PCR (qPCR) of Socs2 transcription in purified HSCs and LSKs from wild-type and *Nras^G12D/+^* mice at a steady state (*n*≥3). (B) Total protein level of Socs2 by western blot in CD48^−^LSKs (HSCs and MPPs) and CD48^+^LSKs (multi-lineage progenitors) from wild-type and *Nras^G12D/+^* mice at steady state. Data are mean±s.d. Unpaired two-tailed Student's *t*-test was used to assess statistical significance. *P≤0.05.

To delineate the mechanism by which N-Ras^G12D^ augments Jak2-Stat5 signaling, we examined key negative regulators of this pathway, including Socs proteins, protein inhibitors of activated STATs (PIASs) and protein tyrosine phosphatases (PTPs). To identify the mechanism by which hyper N-Ras dysregulate HSPCs, we previously performed gene expression profiling with microarray analysis on purified SLAM hematopoietic stem cells (HSCs; CD150^+^CD48^−^Lineage^−^Kit^+^Scal^+^) and multi-potent progenitors (MPP; CD150^−^CD48^−^Lineage^−^Kit^+^Scal^+^) from the *Nras^G12D^* mutant and control mice (Li et al., 2013). In addition, we performed microarray analysis on quiescent (H2B-GFP label retaining) and proliferative (H2B-GFP negative after a 12-week label dilution) SLAM HSCs from the *Nras^G12D^* mutant or control mice that carry *Rosa26-M2-rtTA; Col1A1-H2B-GFP* transgenes (Li et al., 2013). These analyses identified *SOCS2* as one the two genes that met the following criteria: (1) fold increase>2 and *P*<0.05; and (2) consistently dysregulated in all four populations (HSC, MPP, quiescent and proliferative HSC). Other Socs genes or negative regulators of Ras signaling did not meet these criteria (Fig. S4A). Quantitative RT-PCR confirmed the reduction of Socs2 expression in *Nras* mutant HSCs and LSKs (Fig. 2A). Although reduced mRNA levels of *Socs1* and *Socs3* were also detected by qPCR, the difference was much smaller compared with the reduction of Socs2 levels (Fig. S4B). Therefore, we decided to further investigate the role of Socs2 in N-Ras^G12D^-mediated Jak2/Stat5 activation. We first confirmed reduced protein level of Socs2 in *Nras^G12D^* HSCs and early progenitor cells (CD48^−^LSKs) compared with wild-type control (Fig. 2B). Interestingly, although N-Ras^G12D^ and FLT3-ITD both can activate the Stat5 signaling pathway, FLT3-ITD increases Socs2 protein level in progenitor cells (CD48^+^LSK) isolated from FLT3-ITD knock-in mice (Li et al., 2008), while N-Ras^G12D^ reduces Socs2 protein levels (Fig. 2B), suggesting N-Ras^G12D^ and FLT3-ITD have different functions in Socs2 dysregulation.

**Fig. 3.**
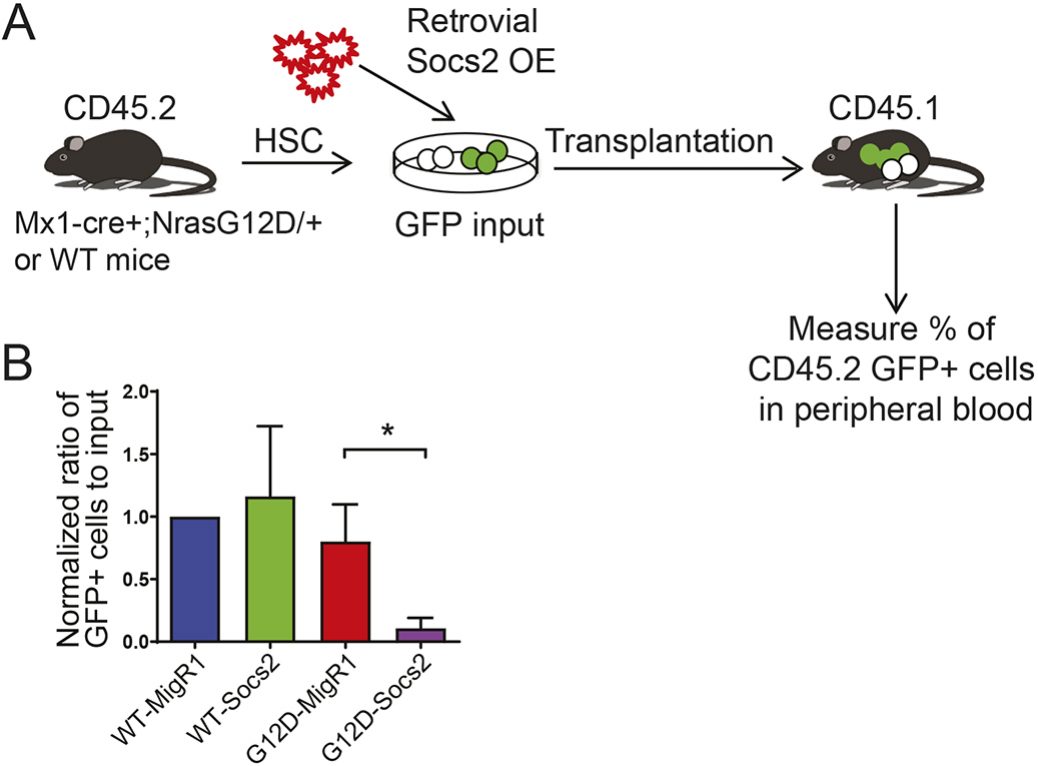
**Restoration of Socs2 expression blocks *Nras^G12D/+^* HSC reconstitution.** (A) Schematic design of overexpressing Socs2 in purified CD45.2 HSCs followed by transplantation into CD45.1 recipient mice (*n*≥14). (B) Normalized ratio of GFP-positive donor percentage to GFP input (% of GFP-positive HSCs before transplantation). Data are mean±s.d. Unpaired two-tailed Student's *t*-test was used to assess statistical significance. *P≤0.05.

Given the role of Socs2 in inhibiting Jak-Stat activation to prevent overt cytokine receptor signaling (Trengove and Ward, 2013), we hypothesized that reduced Socs2 level may contribute to constitutive Stat5 activation and enhanced HSC competitiveness in *Nras^G12D^* HSPCs. To address this, we generated a GFP-expressing retroviral construct that overexpresses the full-length murine *Socs2* gene. Socs2 overexpression was validated in transfected 293T cells (Fig. S5A), and viral titers were determined in transduced NIH3T3 cells (Fig. S5B). Then we transduced purified HSCs from either *Nras* mutant or control mice with empty vector or Socs2OE retrovirus, followed by transplantation into lethally irradiated CD45.1 recipient mice, together with 3×10^5^ CD45.1 wild-type BM cells (Fig. 3A). The percentage of HSCs expressing GFP was determined as GFP input before transplantation (>50% for empty vector; <10% for Socs2OE). At 12 weeks after transplantation, the transplant recipients were bled and the levels of GFP^+^ donor cells were evaluated in peripheral blood by FACS analysis. The percentage of GFP^+^ cells was normalized to the percentage of GFP^+^ donor cells at the time of transplantation to determine the effect of Socs2 overexpression on HSC reconstitution. This showed that Socs2 expression significantly attenuated proliferation/reconstitution of *Nras^G12D^* donor cells, although it had minimal effect on wild-type donor reconstitution (Fig. 3B). These data suggest that N-Ras^G12D^ suppresses Socs2 expression in HSPCs and thus releases the brake on Jak2-Stat5 signaling to promote reconstitution and hyper-proliferation.

### Mutant RAS suppresses SOCS2 expression in human AMLs

**Fig. 4.**
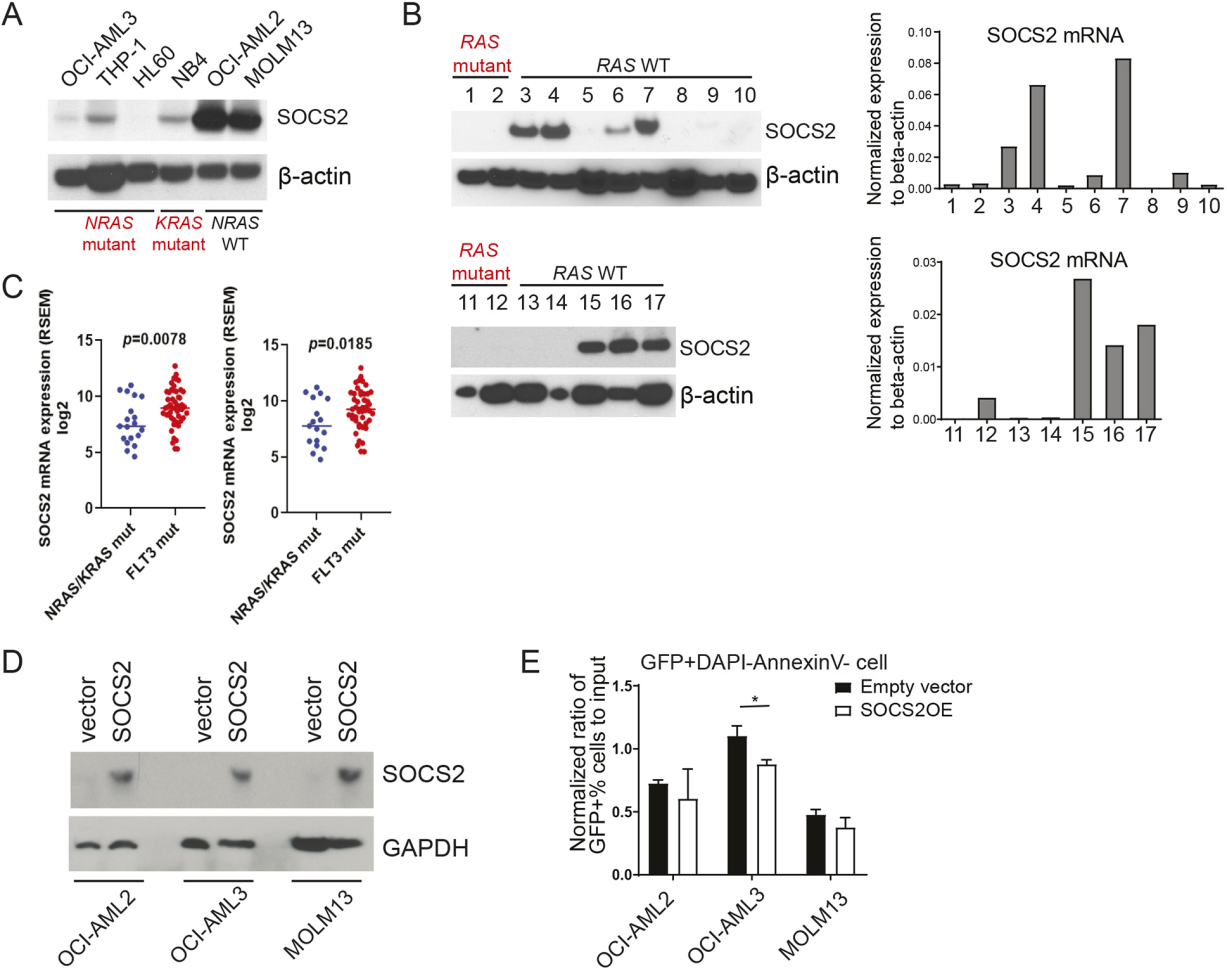
**SOCS2 expression is suppressed and restoration of SOCS2 expression mitigates leukemic growth of RAS mutant AML cells.** (A) Western blot analysis of SOCS2 protein levels in selected *NRAS*-mutant, *KRAS*-mutant and RAS-wild-type AML cell lines. (B) SOCS2 protein and mRNA expression in RAS-mutant and RAS-wild-type primary AML patient samples. (C) *SOCS2* mRNA expression from two independent RNA-seq studies from the public cBioPortal database. (D,E) AML cell lines OCI-AML2 (RAS-wild type, *FLT3*-wild type), OCI-AML3 (*NRAS* mutant) and MOLM13 (RAS-wild type and *FLT3-ITD*) were transduced with a SOCS2-overexpressing retrovirus that co-expresses GFP. (D) Western blot analysis of SOCS2 proteins level in AML cell lines. (E) Percentage of GFP-positive cells were assessed on day 9 by flow cytometry and normalized to the percentage of GFP input on day 0. Data are mean±s.d. Unpaired two-tailed Student's *t*-test was used to assess statistical significance. *P≤0.05.

Accumulating evidence suggests that genetic mutations transform normal HSPCs first into pre-leukemic stem cells and then into leukemic stem cells (LSC) (Notta et al., 2011; Rossi et al., 2008; Welch et al., 2012; Shlush et al., 2014). Our results suggest that oncogenic N-Ras activates Jak2/Stat5 signaling through suppression of Socs2 expression to promote pre-leukemic stem cell proliferation and self-renewal. We next sought to determine whether oncogenic RAS leads to reduced *SOCS2* expression in fully established human leukemia, where RAS mutations are prevalently detected. We performed western blotting in *NRAS* mutant (OCI-AML3, THP-1 and HL60), *KRAS* mutant (NB4) and RAS WT (OCI-AML2 and MOLM13) human AML cell lines, and demonstrated that SOCS2 protein levels were significantly lower in *NRAS* and *KRAS* mutant cells than RAS wild-type AML cells (Fig. 4A). We next examined *SOCS2* expression at mRNA and protein levels in primary blood mononuclear cells purified from AML patient samples (Fig. S6 and Table S1). This showed that RAS mutant AMLs consistently demonstrated significantly reduced levels of SOCS2 (Fig. 4B). Some of the samples without RAS mutations also exhibited loss of SOCS2 expression, suggesting that other genetic mutations may modulate SOCS2 expression in a Ras-dependent or -independent manner.

As *FLT3* is another dominant mutation found in AMLs that leads to the activation of STAT5, we sought to compare SOCS2 expression in *FLT3* and RAS mutant AMLs. By searching the public RNA-seq databases (cBioPortal), we found that *SOCS2* expression in *FLT3* mutant AMLs was significantly higher than RAS mutant AMLs (two independent RNA-seq studies are shown in Fig. 4C). This is also consistent with our findings in pre-leukemic progenitors where Socs2 levels were higher in FLT3-ITD mice (Fig. 2B). Collectively, it suggests that the two mutually exclusive mutations may use different mechanisms to activate STAT5. FLT3-ITD directly acts upstream of STAT5, which subsequently upregulates SOCS2 transcription, while mutant N-RAS suppresses SOCS2, which in turn activates JAK2/STAT5 by removing a negative regulation.

Next, we further assessed how restoration of SOCS2 expression impacts leukemia cell growth by overexpressing SOCS2 in AML cell lines (Fig. 4D). As shown in Fig. 4E, overexpression of SOCS2 only inhibited cell proliferation of AMLs carrying RAS mutations (OCI-AML3) but had minimal effect on *FLT3-ITD* mutant (MOLM13) or RAS and *FLT3* wild-type (OCI-AML2) AML cells. Overall, these data demonstrate that downregulation of SOCS2 is a key mechanism in mutant Ras-mediated abnormalities at both pre-leukemic stem cell and established AML stages.

### Mutant *NRAS* suppresses H3K27 acetylation at the SOCS2 locus in AML cells

**Fig. 5.**
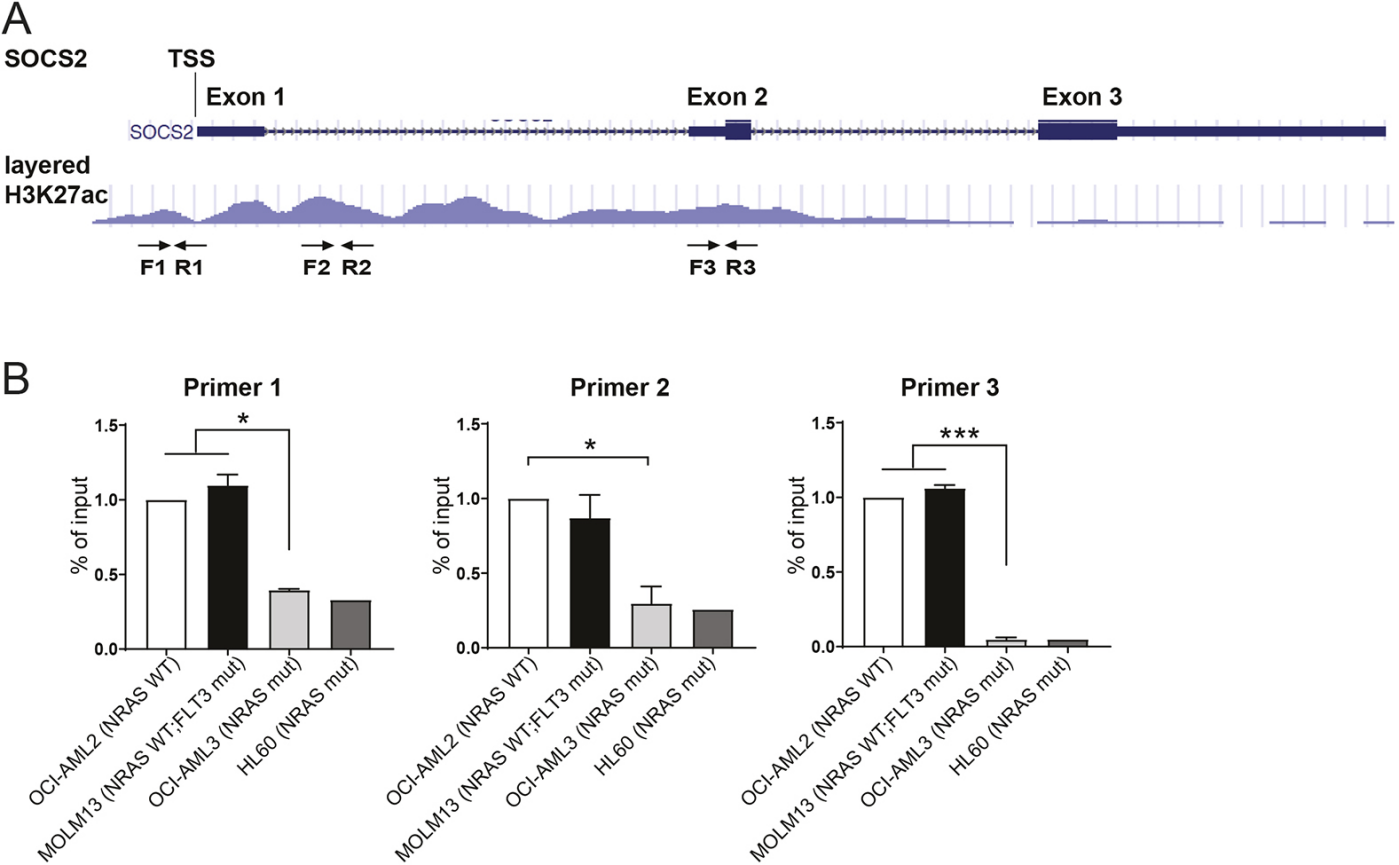
**Mutant N-RAS suppresses H3K27 acetylation at the *SOCS2* locus in AML cells.** (A) H3K27ac-enriched regions at the SOCS2 locus were shown based on the UCSC database. Three sets of qPCR primers are depicted. (B) ChIP-qPCR showing lower H3K27ac levels at the SOCS2 locus in *NRAS*-mutant compared with *NRAS*-WT AML cell lines (*n*=2 for OCI-AML2, MOLM13 and OCI-AML3; *n*=1 for HL60). Data are mean±s.d. Unpaired two-tailed Student's *t*-test was used to assess statistical significance. *P≤0.05, ***P≤0.001.

To understand how *SOCS2* expression is silenced in *NRAS*-mutant AMLs, we hypothesized that epigenetic regulation might be involved. Defined as an active enhancer marker, H3K27 acetylation is usually associated with higher activation of gene transcription (Creyghton et al., 2010). We thus performed chromatin co-immunoprecipitation (ChIP) using H3K27ac antibody, followed by quantitative PCR using primers based on DNA sequence from the *SOCS2* locus. Three primer sets were designed against the regions where H3K27ac is enriched according to the UCSC database (Fig. 5A). For all three regions of *SOCS2* locus we examined, H3K27ac levels were significantly decreased in *NRAS*-mutant AML cell lines OCI-AML3 and HL-60, when compared with RAS wild-type AML lines OCI-AML2 and MOLM13 (Fig. 5B). Because gene expression can be regulated by methylation of the promoters or enhancer elements of the gene, and hypermethylation of *SOCS2* CpG islands was detected in 14% of ovarian cancer patients (Sutherland et al., 2004), we hypothesized that N-Ras^G12D^ may suppress *Socs2* expression by changing methylation of *Socs2* promoter. We therefore performed EpiTYPER/MassARRAY to profile *Socs2* DNA methylation in purified murine LSK cells. This method is based on bisulfide-triggered conversion of non-methylated cytosine to uracil, followed by *in vitro* transcription and detection of target fragments using mass spectrometry. Although *Socs2* promoter displays high GC content, the overall DNA methylation level is very low. Besides, when using primer sets spanning the CpG-island-rich region of the *Socs2* locus (Fig. S7A), we did not observe any differences in DNA methylation between *Nras* mutant and control LSKs (Fig. S7B). These data suggest that *NRAS* mutations alter H3K27ac marks, but not DNA methylation of the promoter, to silence *Socs2* gene expression.

**Fig. 6.**
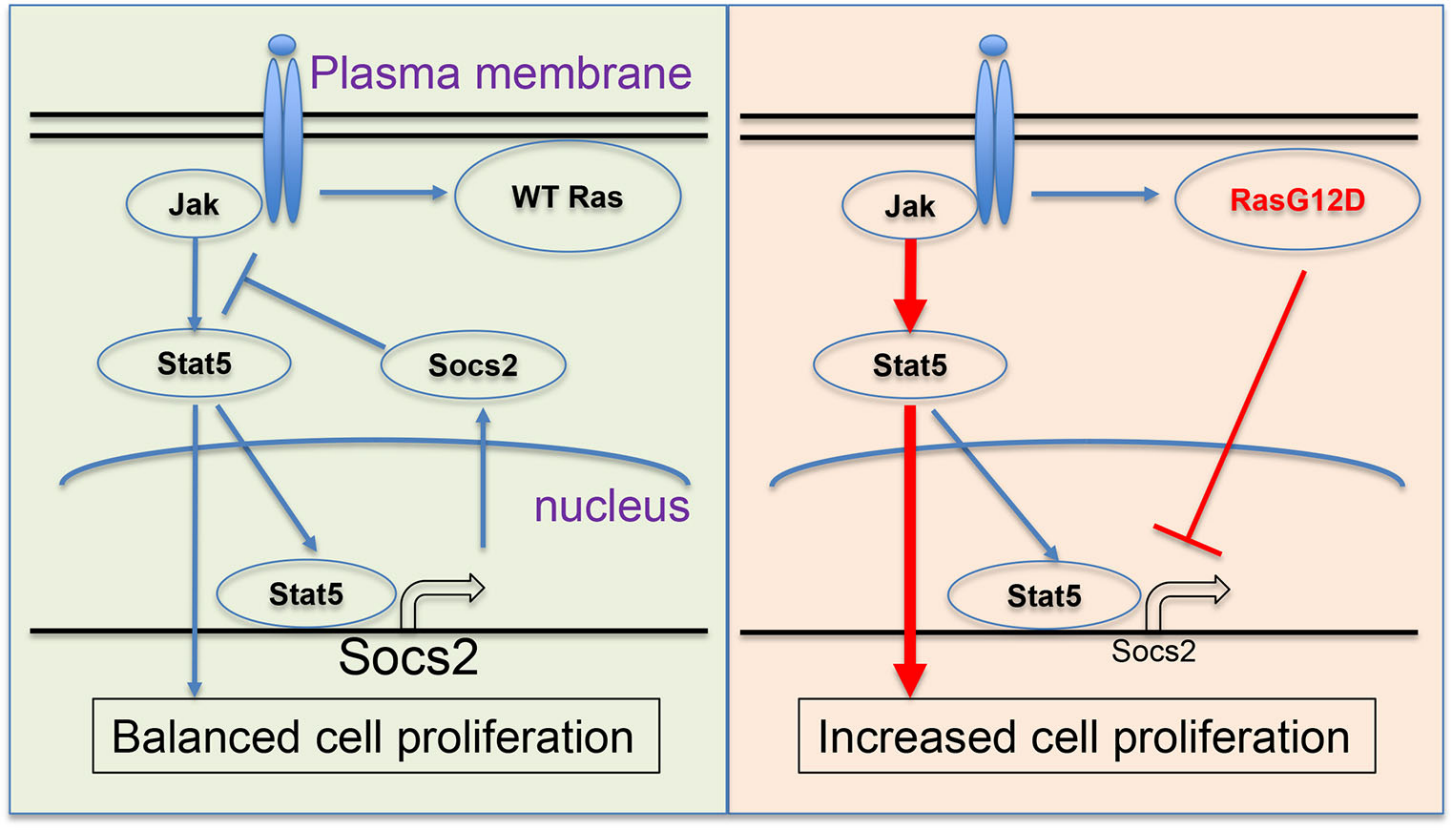
**Schematic working model.** Mutant *Nras* (right) downregulates SOCS2 expression through post-translational modification, therefore enhancing JAK2-STAT5 signaling to promote HSPC expansion and leukemic transformation.

Taken together, we demonstrate that hyperactive N-Ras profoundly suppresses the expression of Socs2, a well-known transcription target and a negative regulator of Jak/Stat, to promote Jak2/Stat5 signaling in HSCs (Fig. 6). This effect likely occurs through modulating the histone H3K27ac marks instead of DNA methylation at the *Socs2* locus. Restoration of Socs2 blocks enhanced HSC competitiveness induced by N-Ras^G12D^, therefore hyperactive N-Ras-mediated pre-leukemic stem cell expansion depends on suppression of Socs2. Decreased levels of SOCS2 are also observed in primary human AMLs and AML cell lines carrying RAS mutations, suggesting that targeting this Ras/Socs2/Jak2/Stat5 signaling axis may have therapeutic implications in AML.

## DISCUSSION

The tight regulation of signaling pathways is pivotal for cells to maintain homeostatic balance to prevent disease initiation. In this study, we report that, while activated Jak/Stat signaling induces transcription target Socs proteins to inhibit Jak/Stat signaling through a negative feedback loop in normal cells, oncogenic N-Ras mutations downregulate Socs2 expression in both mouse pre-leukemic HSPCs and human AML cells, thus leading to hyperactive JAK2-STAT5 signaling and malignant cell expansion.

Stat5 activation underlies key effector cascades in hematopoiesis and leukemic progression (Dorritie et al., 2014). Many genetic mutations have been shown to activate aberrant Stat5 signaling, including FLT3-ITD (Gilliland and Griffin, 2002), BCR/ABL1 (Hansen et al., 2013), JAK2^V617F^ (Ross et al., 2021) and KIT^D816V^ (Baumgartner et al., 2009). Here, we report a novel mechanism by which mutant N-Ras^G12D^ activates Stat5 signaling via a Scos2-Jak2 signaling axis. Interestingly, Socs2 was shown to be dispensable for BCR/ABL1-induced Stat5 activation in a CML model (Hansen et al., 2013). Thus, Socs2-modulated Stat5 dysregulation is unique for oncogenic N-Ras-mediated leukemogenesis.

Socs2 is highly expressed in hematopoietic stem cells compared with more differentiated cell populations (Toren et al., 2005), which suggests an important role in protecting stem cells from aberrant differentiation and proliferation. We demonstrate here that decreased Socs2 level may contribute to enhanced HSC competitiveness and proliferation in the *Nras^G12D^* mutant HSPCs, thus facilitating subsequent leukemia development. In fact, suppression of Socs proteins has been described in many cancer types (Trengove and Ward, 2013), via different mechanisms. For example, although hypermethylation of *SOCS2* CpG islands was detected in 14% of ovarian cancer patients (Sutherland et al., 2004), our studies here revealed that oncogenic N-Ras has minimal impact on DNA methylation of the *Socs2* promoter. Instead, oncogenic N-Ras reduces the active enhancer marker H3K27ac at the *SOCS2* locus, which may explain the lower gene expression of *SOCS2* in *NRAS* mutant leukemia cells. It would be interesting to investigate whether mutant N-Ras exerts this effect through upregulating histone deacetylase (HDAC) activity, as recent studies suggest specific HDACs can drive pathogenesis of blood disorders (Yue et al., 2020).

Unlike mutant N-Ras-mediated Socs2 downregulation, several hematopoietic malignancies driven by other oncogenic mutations demonstrate upregulated *SOCS2* expression, such as JAK2^V617F^-positive essential thrombocythemia (ET) (Puigdecanet et al., 2008), BCR/ABL-positive CMLs (Vitali et al., 2015) and FLT3-ITD AMLs (Janke et al., 2014). In contrast to oncogenic *NRAS* mutations, which indirectly activate Jak2/Stat5 by suppressing *SOCS2*, JAK2^V617F^, BCR/ABL and FLT3-ITD can directly activate Jak-Stat and subsequently induce their transcription target *SOCS2*. These results indicate that dysregulation of SOCS2 plays an important role in oncogenic transformation, but depending on the disease type, upstream mutations and activation mechanisms, SOCS2 can be either activated or inactivated in cancer cells.

Our studies here suggest that activation of Jak-Stat may serve as a universal mechanism through which oncogenic N-Ras dysregulates HSPC function and promote leukemia; therefore, inhibiting Jak/Stat signaling may have therapeutic benefit in RAS mutant cancers. Our previous studies showed that MEK inhibitors alone induce cell cycle arrest in *Nras*-mutant AML; however, no apoptosis of leukemic blasts or cell differentiation was seen (Burgess et al., 2014). In addition, MEK inhibitors rarely lead to sustained clinical benefit in patients (Borthakur et al., 2016; Lauchle et al., 2009; Ney et al., 2020). Our study suggests that combined inhibition of MEK/MAPK and JAK-STAT pathways may provide enhanced efficacy. Consistent with this, a recent report demonstrated that combined treatment with MEK and JAK2 inhibitors significantly prolonged survival in an N-Ras^G12D^-induced CMML (chronic myelomonocytic leukemia) mouse model (Kong et al., 2014). Our study here demonstrates that Socs2 overexpression rescued *Nras^G12D^* HSPC function in bone marrow transplantation; therefore, mechanisms that restore Socs2 expression, perhaps via inhibition of histone deacetylases (HDACs), can rescue Socs2 expression and suppress *NRAS*-mutant leukemic cell growth. In a recent study, a combination of MEK and HDAC inhibitors was shown to attenuate RAS-mutated lung cancer progression through increasing cell apoptosis and cell-cycle arrest (Yamada et al., 2018). Thus, combined inhibition of MEK and HDAC may represent a therapeutic strategy in RAS-driven leukemias.

## MATERIALS AND METHODS

### Animals

The conditional *Mx1-Cre^+^; LSL-Nras^G12D/+^* (Li et al., 2013) and *JAK2^fl/fl^* (Krempler et al., 2004) mouse strains have been previously described. All animals were back-crossed for at least 10 generations onto a C57/BL6 background. Six-week-old mice received intraperitoneal injections of poly (I:C) (GE Healthcare Life Sciences) at a dose of 0.5 μg/g body mass every other day for three doses. Experiments were conducted 2 weeks after poly (I:C) injections. All mice were housed in the Unit for Laboratory Animal Medicine at the University of Michigan, and protocols were approved by the University Committee on the Use and Care of Animals.

### Whole bone marrow transplantation

BM cells from donor CD45.2 mice were transplanted into lethally irradiated CD45.1 recipient mice along with equal numbers of CD45.1 BM cells. Detailed methods can be found in the Supplementary Materials and Methods.

### EpiTYPER/MassARRAY sequencing

Genomic DNA was purified from sorted WT and *Nras^G12D/+^* LSKs (*n*=3). EpiTYPER/MassARRAY sequencing was performed by the University of Michigan Epigenomic Core. In brief, bisulfide triggers conversion of nonmethylated cytosine to uracil, followed by *in vitro* transcription and detection of target fragments using mass spectrometry. Detailed methods can be found in the Supplementary Materials and Methods.

### Antibodies

Antibodies for cell sorting include anti-B220, anti-CD2, anti-CD3, anti-CD5, anti-CD8, anti-Gr-1, anti-CD41, anti-Ter119, anti-CD150, anti-CD48, anti-Kit and anti-Sca1 (Table S2). All antibodies were purchased from BioLegend. Anti-APC conjugated to paramagnetic microbeads were from Miltenyi Biotec. Antibodies for western blotting were anti-pStat5 (Y694), anti-Stat5 (4H1), anti-β-actin (8H10D10), anti-pJak2 (Tyr221), anti-Jak2, anti-pJAK1 (Tyr1034/1035) and anti-SOCS2 (Table S3), all purchased from Cell Signaling Technology. Anti-GAPDH was from Santa Cruz.

### AML cell lines and patient samples

OCI-AML2 and OCI-AML3 were purchased from DSMZ (Germany). THP-1, HL60, NB4 and MOLM13 were kindly provided by Dr Sami Malek (University of Michigan). All cell lines are authenticated and free of contamination. AML patient samples were obtained from the University of Michigan Rogel Cancer Center. Informed consent was obtained from all donors. Clinical investigation was conducted according to the principles expressed in the Declaration of Helsinki.

### Western blotting

10,000 hematopoietic stem cells (HSCs; CD150^+^CD48^−^LSKs), 10,000 multipotent progenitors (MPPs; CD150^+^CD48^+^LSKs), 30,000 CD48^−^LSKs, 30,000 CD48^+^LSKs, 100,000 LSKs (Lineage^−^Sca1^+^cKit^+^) and 1 million whole bone marrow cells were sorted into HBSS with 2% FCS for western blotting. Lineage markers include B220, CD2, CD3, CD5, CD8, Gr-1, CD41 and Ter119. For human AML cells, 1 million cells were used for western blotting. Sample preparation was conducted as previously described (Li et al., 2013).

### Flow cytometry analysis of phospho-STAT5

Bone marrow cells were harvested in IMDM containing 1% BSA, pre-treated with inhibitors at 37°C for 30 min, and then stimulated with Tpo at the concentration of 10 ng/ml for 15 min. Cells were fixed in 2% of paraformaldehyde (Electron Microscopy Sciences), permeabilized in ice-cold 95% methanol and antibody staining carried out as previously described (Jin et al., 2018).

### *In vivo* ruxolitinib treatment and BrdU incorporation

Ruxolitinib (Selleckchem) was administered by oral gavage at 30 mg/kg, twice a day for 7 days. At 24 h before euthanizing the mice, BrdU (Sigma) was intraperitoneally injected as a single dose of 200 mg/kg followed by 1 mg/ml BrdU in the drinking water. Bone marrow cells were saved for western blotting processing. The remaining cells were used for BrdU staining and measurement in gated HSCs and LSKs by flow cytometry (Jin et al., 2018).

### Quantitative real-time RT-PCR

Total RNA was purified using a Qiagen AllPrep Micro Kit (for sorted HSCs and LSKs) or Mini Kit (for human cell lines and primary patient mononuclear cells), reverse transcribed using the High-Capacity cDNA Reverse Transcription Kit (AppliedBiosystems). Quantitative real-time PCR reactions were carried out using SYBR Green MasterMix on an Applied Biosciences qPCR cycler. Relative expression was determined by the Δ/ΔCT method and normalized to the internal control β-actin.

### Retroviral transduction of HSCs and transplantation

Murine Socs cDNA was cloned into the retroviral backbone Migr1 expressing GFP. The Migr1 construct was used as an empty vector control. 96-well plate (V-bottom) were coated with 0.5 µl of Retronectin (0.02 mg/ml, TaKaRa) and left at room temperature for 2 h. 120 μl of alpha-MEM (1% FBS, 1% Glutamine, 50 μM β-ME) containing SCF (100 ng/ml) and TPO (100 ng/ml) was added. One hundred HSCs (CD150^+^CD48^−^LSKs) were sorted into each well and incubated at 37°C for 24 h. Supernatant of empty vector (Migr1) and Socs2OE (pMig-Socs2OE) retrovirus at MOI of 600 (determined by NIH3T3 cells) was added to HSCs, together with 2 μl of 0.1 mg/ml retronectin and 2 μl of 1 mg/ml protamine sulfate. 24 h later, a half volume of the medium was gently replaced with S-Clone SF-O3 (Sanko Junyaku; 1% FBS, 1% glutamine and 50 μM β-ME) containing SCF (50 ng/ml) and TPO (50 ng/ml). This replacement was repeated every 12 h (at least 4 times). On the day of transplantation, 50 μl of CD45.1 whole bone marrow cells (3×10^5^) was mixed with ∼80-100 μl of transduced C45.2 HSCs (one well per mouse). Cells were loaded into an insulin syringe and transplanted into lethally irradiated CD45.1 recipient mice via retro-orbital injection. GFP expression within donor populations was monitored for up to 16 weeks after transplantation.

### ChIP quantitative PCR (ChIP-qPCR)

ChIP was performed in human AML cell lines using Chromatin Immunoprecipitation (ChIP) Assay Kit (Millipore, 17-295). Experiments was carried out according to the manufacturer's instructions. Three sets of qPCR primers were designed against the H3K27ac-enriched SOCS2 locus, according to the UCSC database. Primer sequences were as follows: SOCS2_F1, 5′-GTT CAG TTA GCT CCG CCC AC-3′ and SOCS2_R1, 5′-GGG GGC ATT TCC CAG CTA AC-3′; SOCS2_F2, 5′-AGG ACC TGA TCA AGG GGA CC-3′ and SOCS2_R2, 5′-GAC TGT GGG TCC TCG GAA AC-3′; SOCS2_F3, 5′-TCT CTG CCA CCA TTT CGG AC-3′ and SOCS2_R3, 5′-CGC AGG GTC ATG AGA GAA GG-3′.

### Quantification and statistical analysis

Data are mean±s.d. and were analyzed using unpaired two-tailed Student's *t*-test to assess statistical significance.

## Supplementary Material

Supplementary information
