# Supplementary material for: Epigenetic downregulation of Socs2 contributes to mutant N-Ras-mediated hematopoietic dysregulation
Source: Dis Model Mech. 2022 May 6;15(5):dmm049088. doi: 10.1242/dmm.049088 (PMC9092650; doi:10.1242/dmm.049088)
Supplement: Supplementary information [file dmm-15-049088-s1.pdf]

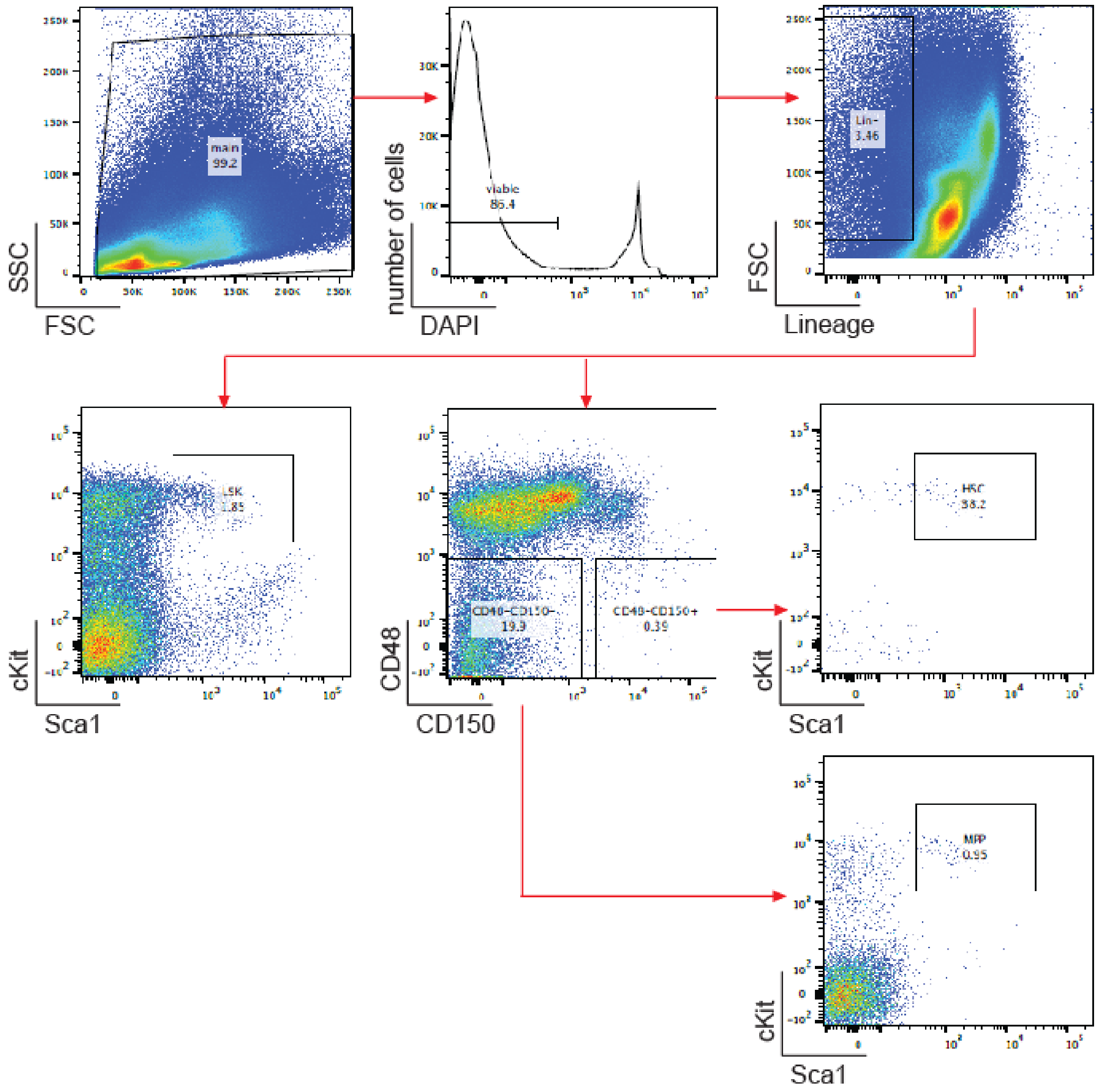

Fig. S1. Gating strategy to either sort or analyze murine hematopoietic stem cells by flow cytometry. HSC: $CD150^{+}CD48^{-}Lineage^{-}Sca1^{+}cKit^{+}$; MPP: multipotent progenitors, $CD150^{-}CD48^{-}Lineage^{-}Sca1^{+}cKit^{+}$; LSK: larger pool of primitive progenitors and HSCs, $Lineage^{-}Sca1^{+}cKit^{+}$. Viable cells are gated as DAPI staining negative cells.

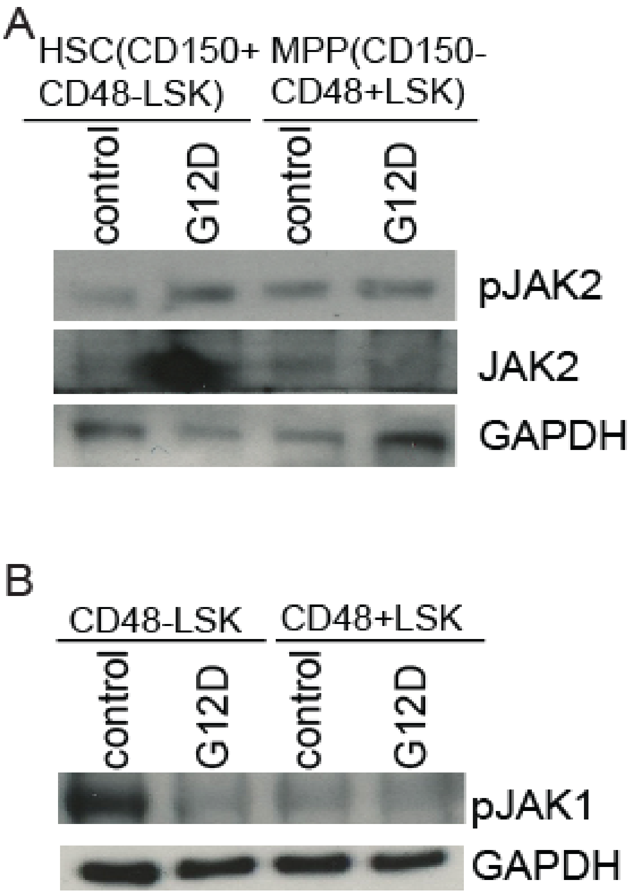

Fig. S2. Additional blots for JAK activity in N-Ras^{G12D} HSPCs. (A) Western blot analysis of pJak2 at steady state in primitive HSCs and MPPs. **(B)** Western blot analysis of pJak1 at steady state in CD48^{-}LSKs (HSCs and MPPs) and CD48^{+}LSKs (multi-lineage progenitors).

Disease Models & Mechanisms • Supplementary information

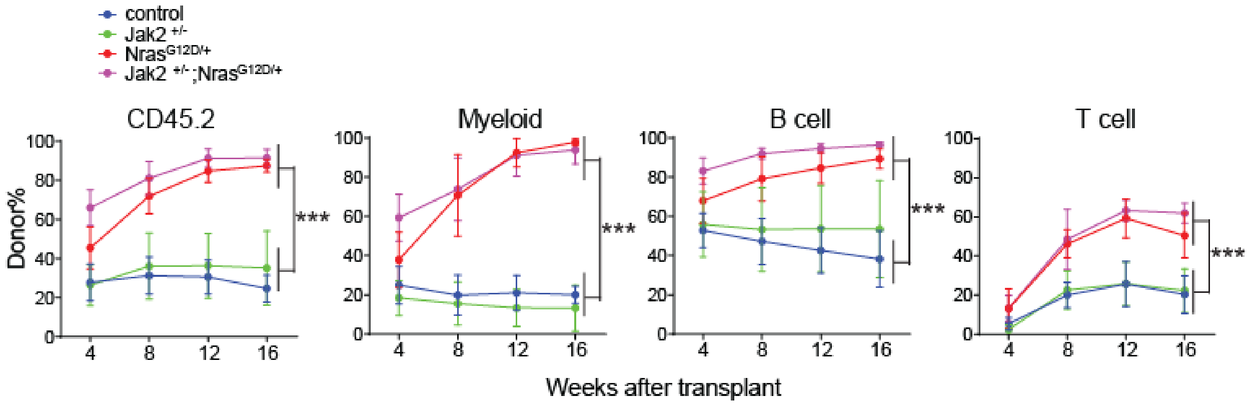

Fig. S3. Heterozygous deletion of Jak2 has no effect on N-Ras^{G12D}-induced HSPC competitiveness and self-renewal. Donor (CD45.2) bone marrow cells ($5x10^5$) from WT, Nras$^{G12D/+}$, Jak2$^{+/-}$ and Nras$^{G12D/+}$;Jak2$^{+/-}$ mice at 2 weeks after poly (I:C) treatment were transplanted into lethally irradiated recipient (CD45.1) mice along with $5x10^5$ CD45.1 bone marrow cells. Donor-cell reconstitution (CD45.2) in total nucleated cells, myeloid (Mac1$^+$Gr1^{low} and Mac1$^+$Gr1$^+$), B- (B220$^+$) and T-cell (CD3$^+$) lineages was assessed in peripheral blood for 20 weeks after transplantation. Data represent mean ± SD. Two-tailed Student *t* tests were used to assess statistical significance. *P < .05, **P < .01, ***P< .01.

Disease Models & Mechanisms • Supplementary information

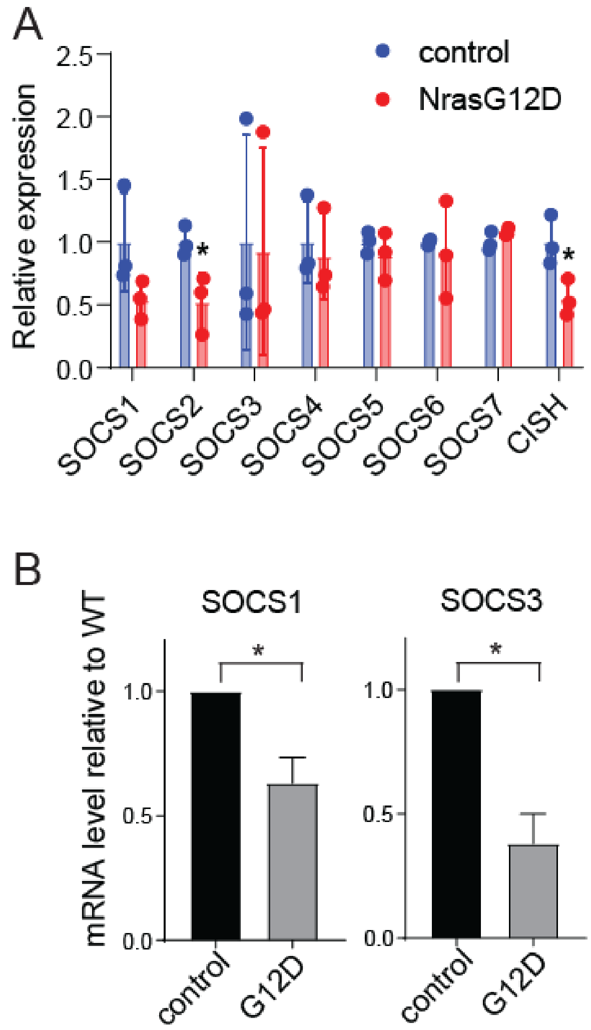

Fig. S4. Gene expression of SOCS family members in HSCs. (A) Levels of SOCS genes from Microarray analysis comparing control and *Nras*^{G12D} HSCs (n=3). Representative probe with the highest signal for each gene is shown. **(B)** Quantitative RT-PCR (qPCR) of SOCS1 and 3 transcription in purified HSCs from control and *Nras*^{G12D} mice (n≥3). Data represent mean ± SD. Two-tailed Student t tests were used to assess statistical significance. *P ≤ .05, **P ≤ .01.

Disease Models & Mechanisms • Supplementary information

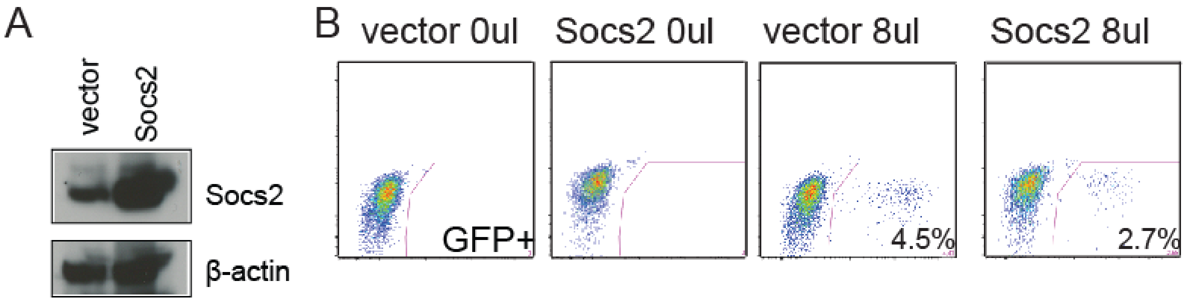

Fig. S5. Retro-viral overexpression of Socs2. (A) Overexpression of Socs2 was confirmed in transfected 293T cells by Western blotting. **(B)** Viral titration (determination of MOI) of empty vector and Socs2OE viral supernatant in NIH3T3 cells. Flow cytometry showed robust GFP expression, suggesting successful infection of cells to overexpress empty vector and Socs2.

Disease Models & Mechanisms • Supplementary information

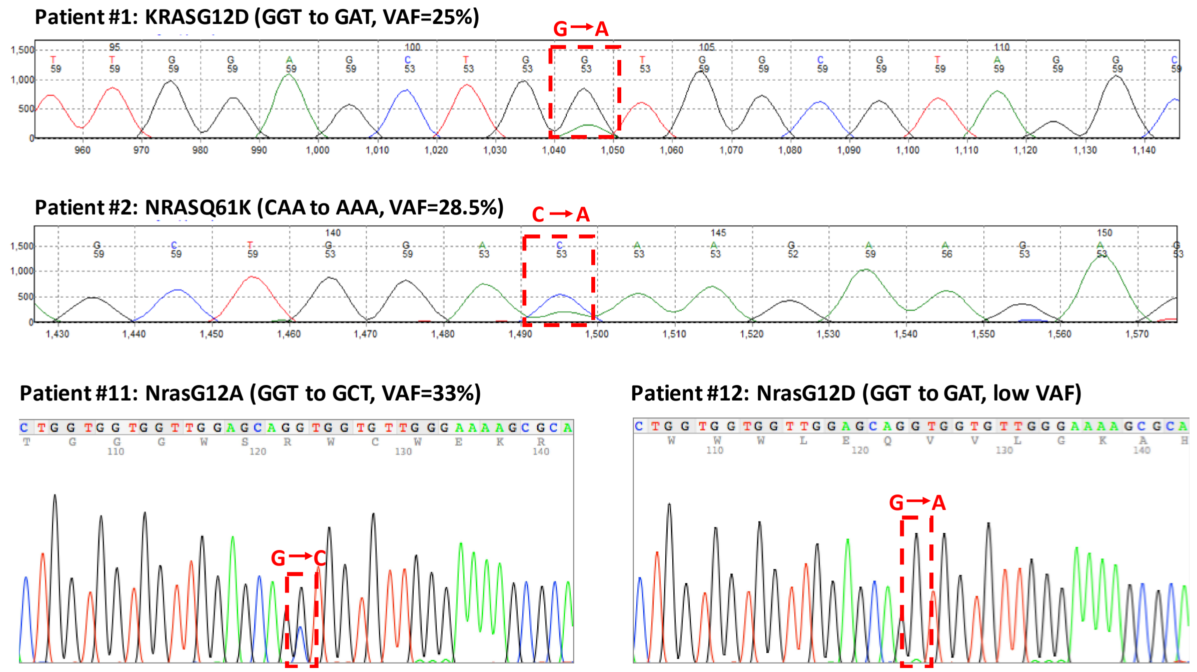

Fig. S6. RAS variant allele frequency (VAF) in AML samples used in this study.

Disease Models & Mechanisms • Supplementary information

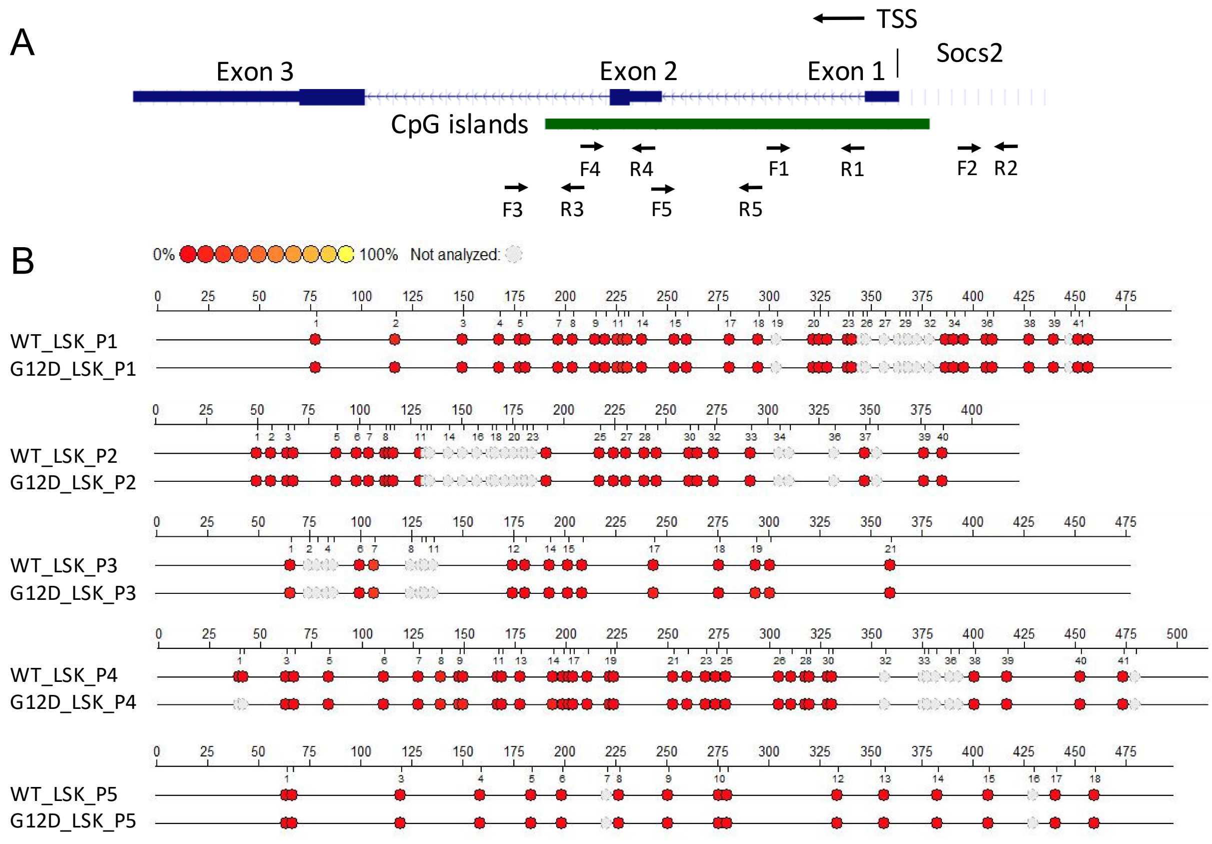

Fig. S7. N-Ras^{G12D} has no effect on altering *Socs2* DNA methylation in LSKs. (A) The promoter region and putative CpG island of *Socs2* gene are depicted according to the UCSC database. Five sets of primers were designed and used for PCR amplification to cover the promoter and CpG island. **(B)** Representative plots of DNA methylation by EpiTYPER/MassARRAY sequencing. LSKs were sorted and pooled from WT and N-Ras^{G12D} mice.

Disease Models & Mechanisms • Supplementary information

Table S1. AML genetic mutations

Patient ID	*Genetic mutations	RAS Variant allele frequency (VAF)	Bone marrow aspirate blast (%)
1	KRASG12D	25% (GGT to GAT)	62.4
2	NRASQ61K	28.5% (CAA to AAA)	70
3	FLT3, NPM1		74.8
4	None		56.4
5	NPM1, IDH1/2		N/A
6	FLT3, NPM1		N/A
7	FLT3		N/A
8	FLT3, NPM1		66.6
9	FLT3, IDH1/2		89.6
10	IDH1/2		81.2
11	NRASG12A	33% (GGT to GCT)	N/A
12	NRASG12D, FLT3ITD	low (GGT to GAT)	41.8
13	IDH2 exon 4		88
14	NPM1		N/A
15	IDH1 exon 4, #JAK2V617F		47.7
16	FLT3D835, NPM1		97
17	FLT3ITD, NPM1		N/A

* The mutation screening panel includes genes *NRAS, KRAS, FLT3, NPM1, CEBPα, IDH1/2 exon 4, cKIT, TET2, GATA2* and *RUNX1*

JAK2V617F mutation was reported at diagnosis, but was not included in the screening panel for other patient samples

Disease Models & Mechanisms • Supplementary information

Table S2. Antibodies for cell sorting

FACS antibody					
Antigen	Conjugate	Cat#	Dilution (Folds)	Clone	Company
Gr-1	FITC	108406	800	RB6-8C5	Biolegend
B220	FITC	103206	200	RA3-6B2	Biolegend
CD2	FITC	100105	200	RM2-5	Biolegend
CD3	FITC	100206	200	17A2	Biolegend
CD5	FITC	100606	200	53-7.3	Biolegend
CD8	FITC	100706	200	53-6.7	Biolegend
TER-119	FITC	116206	200	TER-119	Biolegend
c-Kit	APC	105812	200	2B8	Biolegend
Sca-1	PERCP	108124	200	D7	Biolegend
CD48	PECY7	103424	200	HM48-1	Biolegend
CD150	PE	105904	100	TC15-12F12.2	Biolegend
CD45.1	Brilliant Violet 605	110738	200	A20	Biolegend
CD45.2	Alexa Fluor 700	109822	200	A20	Biolegend
Annexin V	APC	BDB550474	40		BD

Table S3. Antibodies for western blotting

Western Blot antibody			
Ab	Cat#	Dilution	Company
Phospho-Stat5 (Tyr694) Antibody	#9351	1000	Cell Signaling Technology
Stat5a (4H1) Mouse mAb	#4807	1000	Cell Signaling Technology
Socs2	#2779	1000	Cell Signaling Technology
β-Actin (8H10D10) Mouse mAb	#3700	1000	Cell Signaling Technology
Phospho-Jak2 (Tyr221) Antibody	#3774	1000	Cell Signaling Technology
Phospho-Jak1 (Tyr1034/1035) Antibody	#3331	1000	Cell Signaling Technology
GAPDH	sc-25778	1000	Santa Cruz Biotechbology

Disease Models & Mechanisms • Supplementary information

Supplementary Materials and Methods

Bone marrow transplantation

For long-term competitive repopulation assay, $5x10^5$ of donor bone marrow cells from WT, $Nras^{G12D/+}$, $Jak2^{+/-}$ and $Nras^{G12D/+}$;$Jak2^{+/-}$ mice were transplanted along with $5x10^5$ competing bone marrow cells (CD45.1) into lethally irradiated (2x550 Rad) CD45.1 recipient mice by retro-orbital injections. Donor engraftment was monitored for 20 weeks after transplantation with blood staining.

EpiTYPER/MassARRAY sequencing

Genomic DNA was purified from sorted WT and $Nras^{G12D/+}$ LSKs (n=3). EpiTYPER/MassARRAY sequencing was performed by University of Michigan Epigenomic Core[1]. PCR primer sequences are listed as below: Socs2_F1, 5’- aggaagagagATGGATAGGTTTTTGTTATGGGTAA -3’ and Socs2_R1, 5’- cagtaatacgactcactatagggagaaggctAAAACCTCAATCCCAATTAATAACA -3’; Socs2_F2, 5’- aggaagagagGAGGAAAGGTTTATGTTTTAGGGG -3’ and Socs2_R2, 5’- cagtaatacgactcactatagggagaaggctCATTAACTAAAAACTTCTCCTTATTCCC -3’; Socs2_F3, 5’- aggaagagagGGGTTTATTTTGGAAATGTTTTTTT- 3’ and Socs2_R3, 5’- cagtaatacgactcactatagggagaaggctAAAAAACTAAAACTTTTTCTCCTCTCC -3’; Socs2_F4, 5’- aggaagagagTGGGGTATTTTGTGATTTTTAGGT -3’ and Socs2_R4, 5’- cagtaatacgactcactatagggagaaggctAAACATTTCCAAAATAAACCCAACT -3’; Socs2_F5, 5’- aggaagagagGTAGGTGATTGTTTTTTGGGTTG -3’ and Socs2_R5, 5’- cagtaatacgactcactatagggagaaggctTACCTAAAAATCACAAAATACCCCA -3’.

Reference:

1. Quantitative high-throughput analysis of DNA methylation patterns by base-specific cleavage and mass spectrometry. PNAS 2005. doi/10.1073/pnas.0507816102
